# Some Comments upon Erichsen’s “Concussion of the Spine”

**Published:** 1895-01

**Authors:** S. C. Red

**Affiliations:** Houston, Texas


For Texas Medical Journal.

### SOME COMMENTS UPON ERICHSEN'S "CONCUSSION OF THE SPINE."

BY S. C. RED, M. D., HOUSTON, TEXAS.

[Read before the Houston District Medical Society.]

THIS little work of fourteen chapters, written in 1866, and re-written in 1882, contains in its few short pages matter of no little interest. The cause of this unusual prominence or interest is due chiefly to the important relation it bears to suits for damages against railroads. It has been the chief actor in these suits so often that many have come to regard it as "Erichsen on Railway Spine," instead of "Concussion of the Spine, by Erichsen." In fact, the work is little known and discussed outside of railway circles. Here, however, it has come in for the anathema of not a few chief surgeons and many subordinate ones.

They recognize in it the means of furnishing to unscrupulous men abundant opportunity to become most accomplished malingerers, for Mr. Erichsen wields a facile pen. He is a bold and vivid picturer of symptoms, a concise and vigorous writer.

With all his brilliancy, however, he has fallen into some errors, plain to the casual observer. For example: Anatomists define the spine as a column of flexible bones, called vertebras, yet Mr. Erichsen speaks in his very title of concussing this column of bones. To the average medical mind, as well as to the extraordinary one, concussion applies to "An organ, as concussion of the brain." (Dungluson.)

His statement that he follows in the wake of previous writers, and uses their phraseology, is no excuse for a teacher, and one that poses as a scientist. If such men do not use technically correct language, of whom are we to expect it?

If you will pardon the presumption of one who has carefully read his work, I might suggest as a title, "Concussion, compression, and cotusion of the back." This comes nearer expressing what he decribes in his work. Why he chose such an unscientific and unnatural name, has no satisfactory explanation. He has, I believe, recently suggested "Traumatic Neurosis" as a name for his work, but that even is unsatisfactory, since it covers only a fraction of his cases, and, for the others, names a symptom as the disease.

In his collection of fifty-three peculiar cases, I defy any one to point out more than 25 per cent that can be strictly classed as concussion of the spinal cord. I might even go so far as to say 10 per cent, or even fewer, can not be so classed. That such a condition exists, no one questions, but that spinal concussion (so-called) is rare, I am prepared to defend by his own publication.

Even though Mr. Erichsen claims that railways have greatly increased the affection, yet when he quotes from the records of our civil war, he states, "That 75 cases are attributed to contusions and miscellaneous injuries of the spine." When we consider the countless injuries that were inflicted during that fratricidal war, and that only 75 were reported as being spinal (dislocations and fractures excluded), it is at once patent, the rarity of this affection. Unfortunately for Mr. Erichsen's statement that injuries of the spine have greatly increased since the advent of railways, only two are attributed to that cause.

Still further as to its rarity, even in his fifty-three published cases, you are at times seriously in doubt but that Mr. Erichsen has improperly diagnosed the affection and should have called it cerebral instead of spinal. Not a few of his cases point to compression or contusion. When you consider the anatomy of the part, viz.: an ounce of spinal cord in a hydrostatic tube, surrounded by bone, ligament, muscle, fat and skin, great is the wonder that it is ever injured. Another point I wish to make against Mr. Erichsen is, that he is credulous, consequently everything that he says cannot be accepted as law and gospel. He takes as true that a patient suffering from pareoplegia of several months standing, had not lost the "normal physical development of his limbs." He not only accepts it as true, but comments on it to the effect that wasting of the tissue must not be considered as necessary to paralysis. The general contour of the limbs may have remained in statu quo, due to a deposit of fat, but for them to retain their "normal physical development," I am satisfied that no one in this audience is prepared to accept unquestioned. To illustrate further his credulity, he comments approvingly on the case of a woman who had a temperature, for three months, ranging from 107° to 122°.

This brings to my mind, vividly, the experience of Dr. DaCosta. One of his female patients kept a ridiculously high temperature, varying at all unseasonable hours in a remarkable manner. The nurse at last detected her heating the thermometer in the candle. After that the temperature registered normal.

This case is very much like what we read in medical journals. And the profession has grown quite skeptical about journal cases.

Mr. Erichsen has further found a condition or state the like of which I have not met. He claims that railway injuries of the spine are peculiar but not different from those produced by other causes; a distinction without a difference. He thinks this peculiarity is due to an undescribed thrill or vibration that accompanies the accident. He leaves the impression that he intends to say: that when the train is wrecked, the whole mass and its contents quiver like the free end of an arrow when stuck in hard wood. I have seen a good many men who have been in wrecks and they all say, "It is done so quickly we do not know anything about it." They have no recollection of a thrill or vibration.

This thrill business is like another instance of the author's credulity. Some nervous female has been imposing upon him.

My belief is, that, taking the same force, the result will be the same, no matter what the cause. It is a matter of common observation that railway injuries are more extensive than appears from external observation of the tissues affected. It is a practice to amputate higher up than the line of injury would indicate. This condition, if I may hazard an opinion, is brought about by reason of the great force of the causative agent, and not from any peculiarity in the same.

Mr. Erichsen has not satisfactorily digested the subject of definition. First, he claims that concussion of the spine embraces injuries to the bones, ligaments, muscles of the back and spinal cord, yet, in his attempt at definition, the injury is limited to the cord. He defines S. C. as follows: "A certain state of the spinal cord, occasioned by external violence; a state that is independent of, and usually, but not necessarily uncomplicated by any obvious lesion of the vertebral column, such as its fracture or dislocation,—a condition that is supposed to depend upon a shake or jar received by the cord, and in consequence of which its intimate organic structure may be more or less deranged, so that various symptoms indicative of loss or modification of inervation are immediately or remotely induced." This definition is rather ambiguous. When shorn of all superfluities, it may be stated as a shake or jar received by the cord, giving rise to structural change.

The natural tendency of concussion is to recovery, and only occasionally do structural changes result. This is the opinion expressed by Ashurst, and, according to my limited experience, is correct. Three cases have come under my observation out of an hospital experience covering twenty thousand cases among railway employes, and all of these recovered, with one exception. This exception is in an hysterical female who is more liable to attacks of hysteria than formerly. I will ask your indulgence again at my temerity in attempting a definition in which Mr. Erichsen has so signally failed, viz.: A shake or jar of the spinal cord giving rise to functional changes which may be followed by alterations in structure. This definition is not intended to embrace the traumatic neurosis treated so extensively in his work; for example, he relates a case of a man who suffered from a contused wound of the finger caused by the closing of a car door. As a result of this contusion he ultimately suffered from serious central lesions of the nervous system. This case is, indeed, a curiosity; such lesions do not ordinarily result from so simple an injury.

Now, if it is curiosities he wishes to collect, we all can multiply them. I, even, can cite a case of a man out west whose death was caused by the bite of a fly. His classification of tetanus as a traumatic neurosis is pardonable from the fact that it was so understood and taught at the time of writing his book.

One general charge I wish to bring against the work is, that it is not up to the general standard of scientific works. No one man can, however, accomplish everything; as the author states, it is a class of injuries not thoroughly understood. He gives a history out of his large experience of quite a number of peculiar cases, delineating their symptoms in a clear and forcible manner.

A study of this work puts the surgeon on his guard against making any kind of prognosis in injuries of the nervous system.

It better equips him to recognize obscure injuries, and furnishes him a rational plan of treatment. His chapter on symptoms will equal any by our classical authors. It is clear, logical and forcible in its delineation of signs and symptoms. Hardly a line but is full of interest to the seeker after knowledge.

These pages teach pleasantly a hard lesson in surgery, and make one thankful for the genius that worked it out. While it is not perfect, yet it will be a fitting monument to the labors of a great man.

I desire now to report the case of a man who consulted me often after I had written this paper. He presents an array of symptoms apparently copied from Erichsen. I will make no comments, and leave you to draw your own conclusions: A. M., 40 years, looked 55, who, three years ago, was so severely affected by an acute pain in the upper dorsal region that he was confined to his bed for two weeks. He does not remember any paralysis, although he was helpless in bed. There were no external evidences of injury, not even a red spot. After recovering from the acute symptoms, he essayed to return to work, but found himself unfit for duty, not that he felt any particular pain or sickness, but was just unfit for his work as a land agent. Then he noticed that he did not sleep as well as usual. This symptom has gradually grown worse, until now his troubled sleep will not last over two hours without an anodyne. His face flushes from no reason whatever. He hears strange sounds. His vision is not so good as it was. He dreads to meet people in the street, even though he is a land agent. He has continual occipital headaches. There are painful spots on his spine, particularly over the point of first lesion, viz.: upper dorsal region. Girdle pains are continually present. From a large robust man of 185 pounds he has fallen away to 130 pounds. In fact, he and his friends notice that he is a changed man. All this came from sleeping on a hard place in his bed. He has consulted many eminent men, but in vain—and now, the burden of his life is:

The only thing that makes me rue it
'Twant a railroad so 's to sue it.
They gave me arsenic pellets, pills,
To tame my curious spinal ills.
Strychnine? Yes sir—by the pound
For those pains that girdle round.
I'm burnt from occiput to coccyx,
That left me surely in a fix.
One big doctor said I was insane—
That wheels I must have in my brain.
One filled my eyes with iodides,
Some others gave me the bromides.
In fact they gave drugs galore
That heaven or earth has ever bore.
The 'lectric current made me quake;
The patent medicine and the fake
Had a trial, but in vain—
Still round my innards there is pain.
I'm getting worse instead of better—
Its nature now, I'm going to let her
Have a trial, she can do as well.
The doctors so far have played h—!
The only thing that makes me rue it,
'Twant a railroad so 's to sue it.

